# Supplementary material for: Phylogenetic proximity is a key driver of temporal succession of marine giant viruses in a five-year metagenomic time-series
Source: ISME Commun. 2025 Nov 21;5(1):ycaf217. doi: 10.1093/ismeco/ycaf217 (PMC12694412; doi:10.1093/ismeco/ycaf217)
Supplement: Supplemental_data_ycaf217 [file supplemental_data_ycaf217.docx]

Supplemental file for: Phylogenetic proximity drives temporal succession of marine giant viruses in a five-year metagenomic time-series


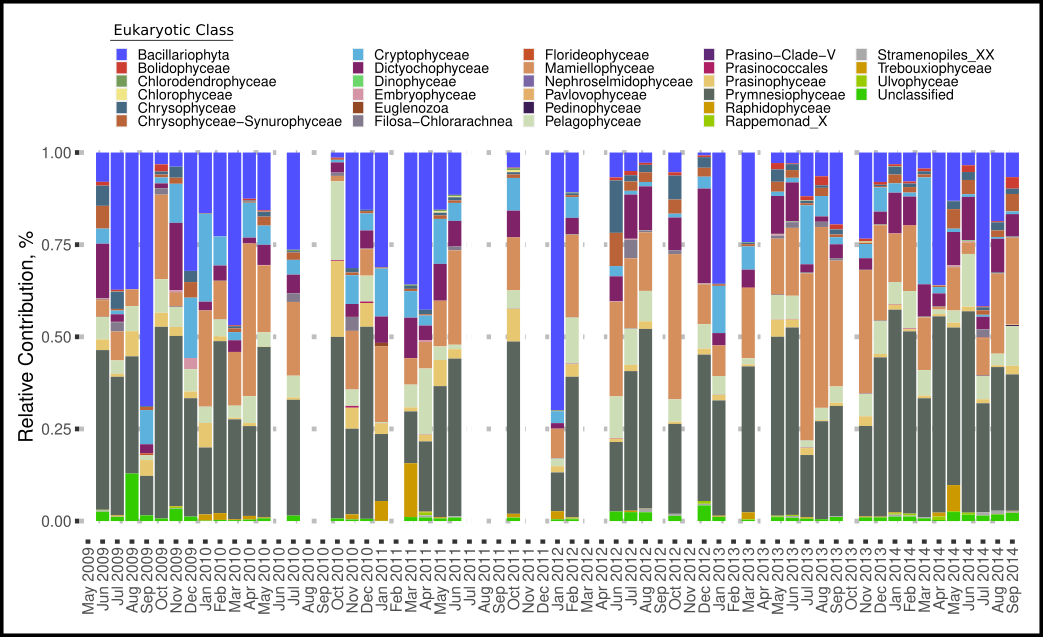


**Figure S1.** Eukaryotic diversity recovered from chloroplast 16S. The relative abundance of Eukaryotic OTUs classified to the class level is shown here.


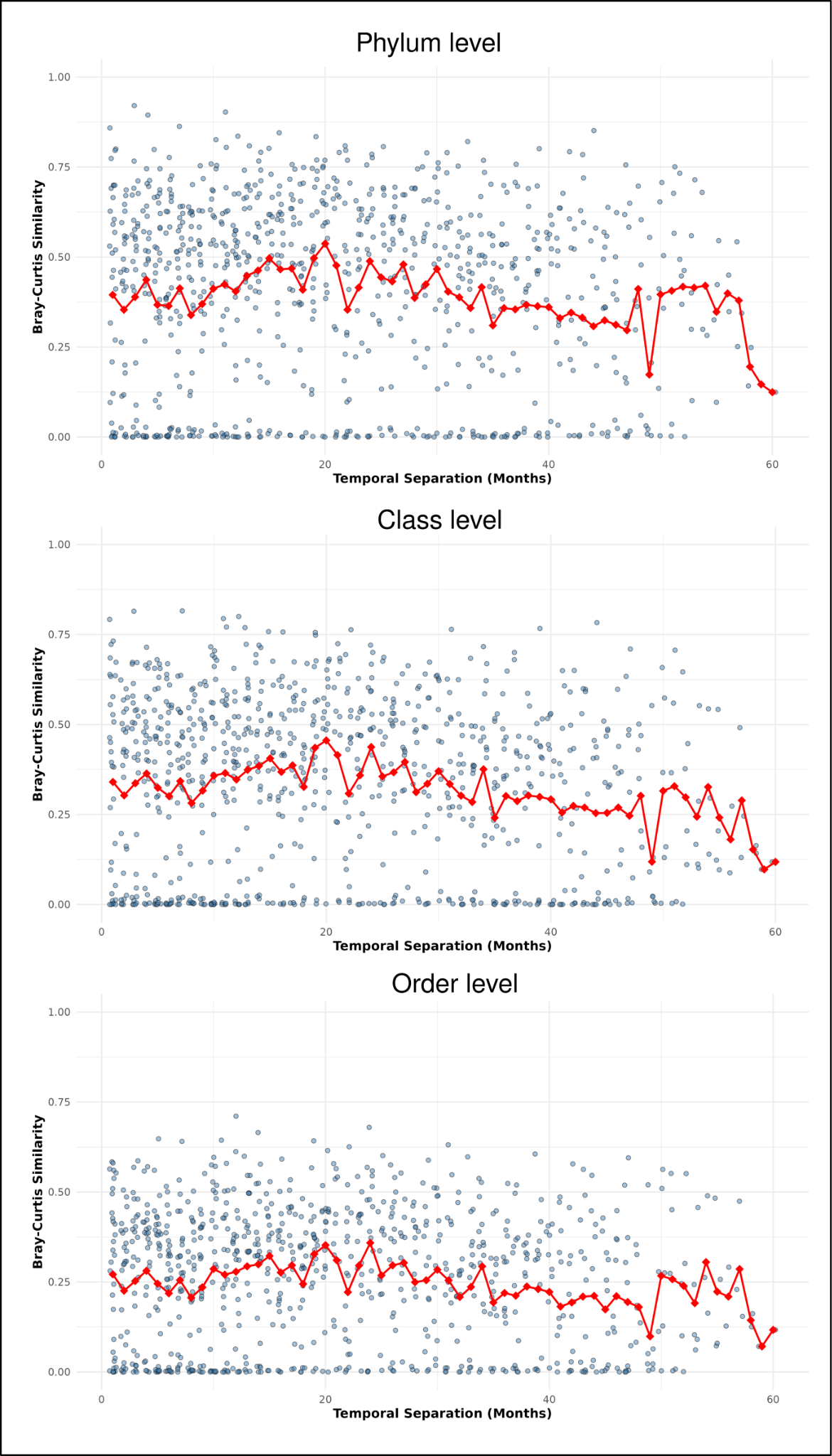


**Figure S2. Bray-Curtis similarity in the eukaryotic community at different taxonomic levels.** Bray-Curtis similarity was calculated using summed abundance values from three different levels of taxonomy (phylum, class, and family).


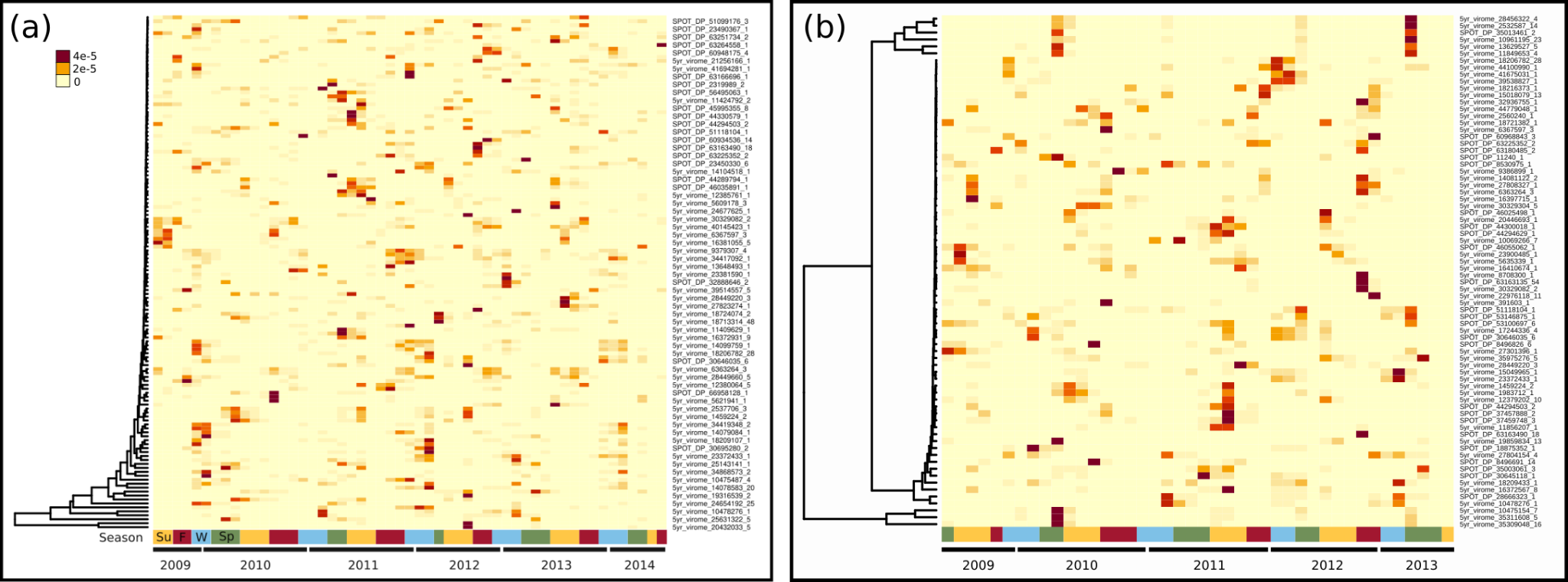


**Figure S3. Seasonality of NCLDV phylotypes.** Normalized abundance of the NCLDV PolB phylotypes with no significant seasonality using the extended Fisher’s g test is shown in both the (a) viral and (b) cellular fraction. Phylotypes were clustered based on hierarchical clustering of abundance patterns.


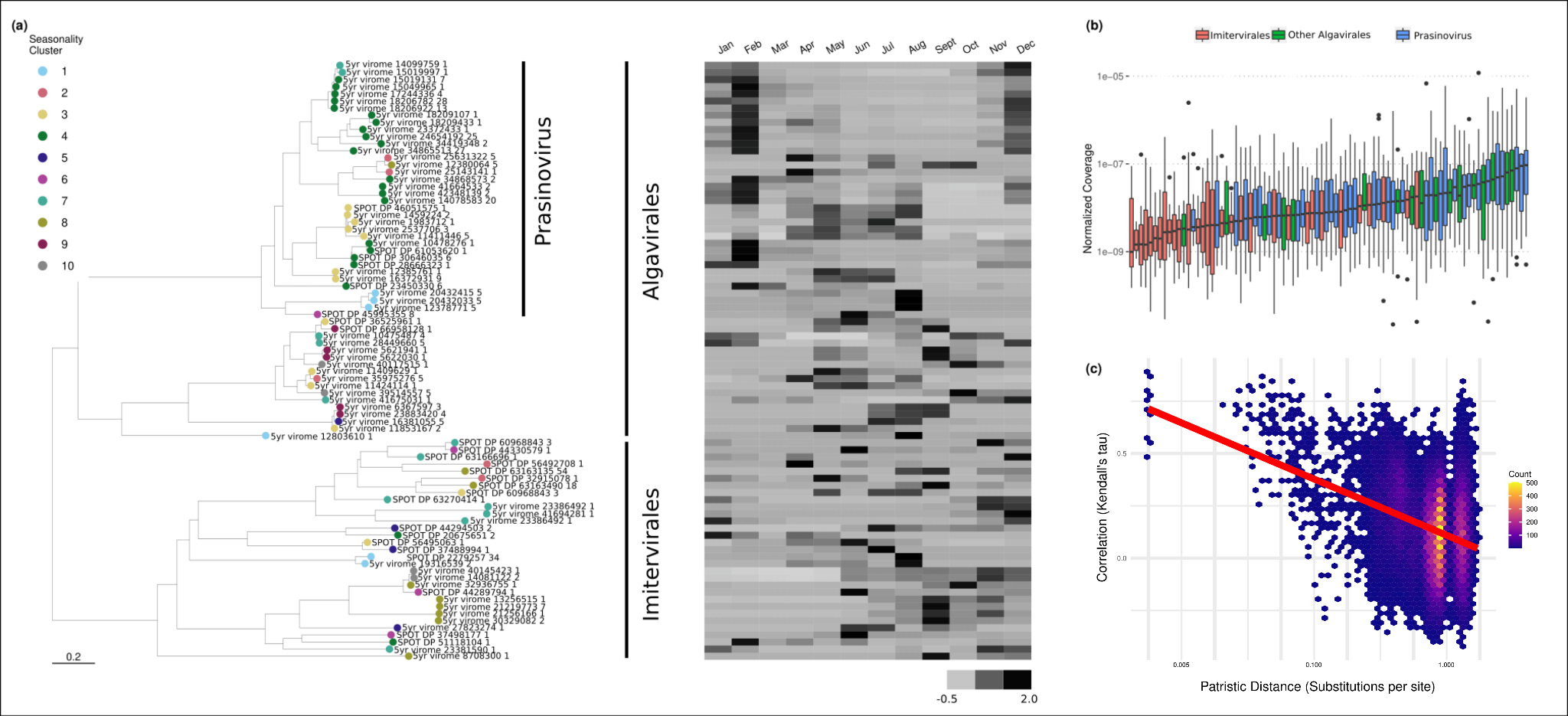


**Figure S4. Expanded figure 4.** Replica of figure 4 with leaf labels added to the phylogenetic tree.


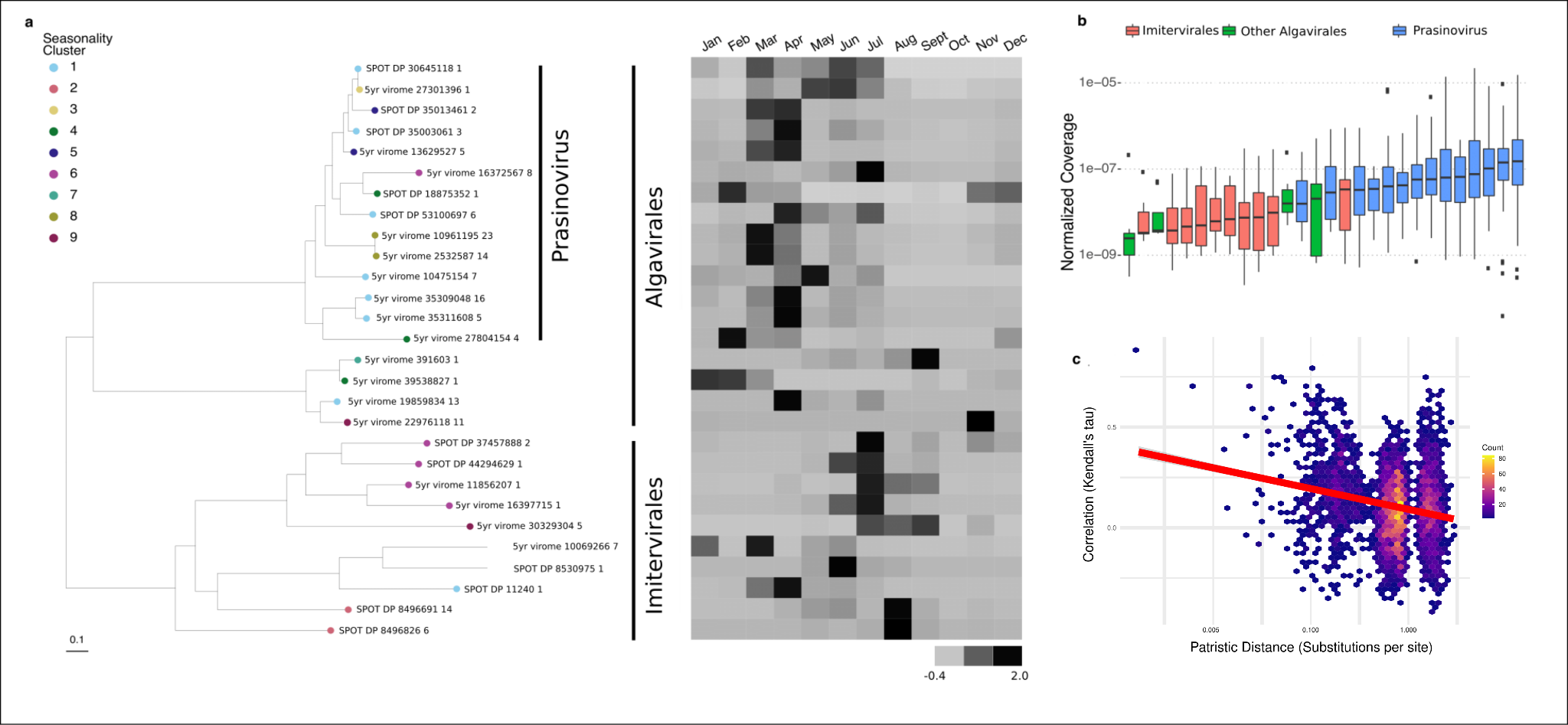


**Figure S5. Expanded figure 5.** Replica of figure 5 with leaf labels added to the phylogenetic tree.


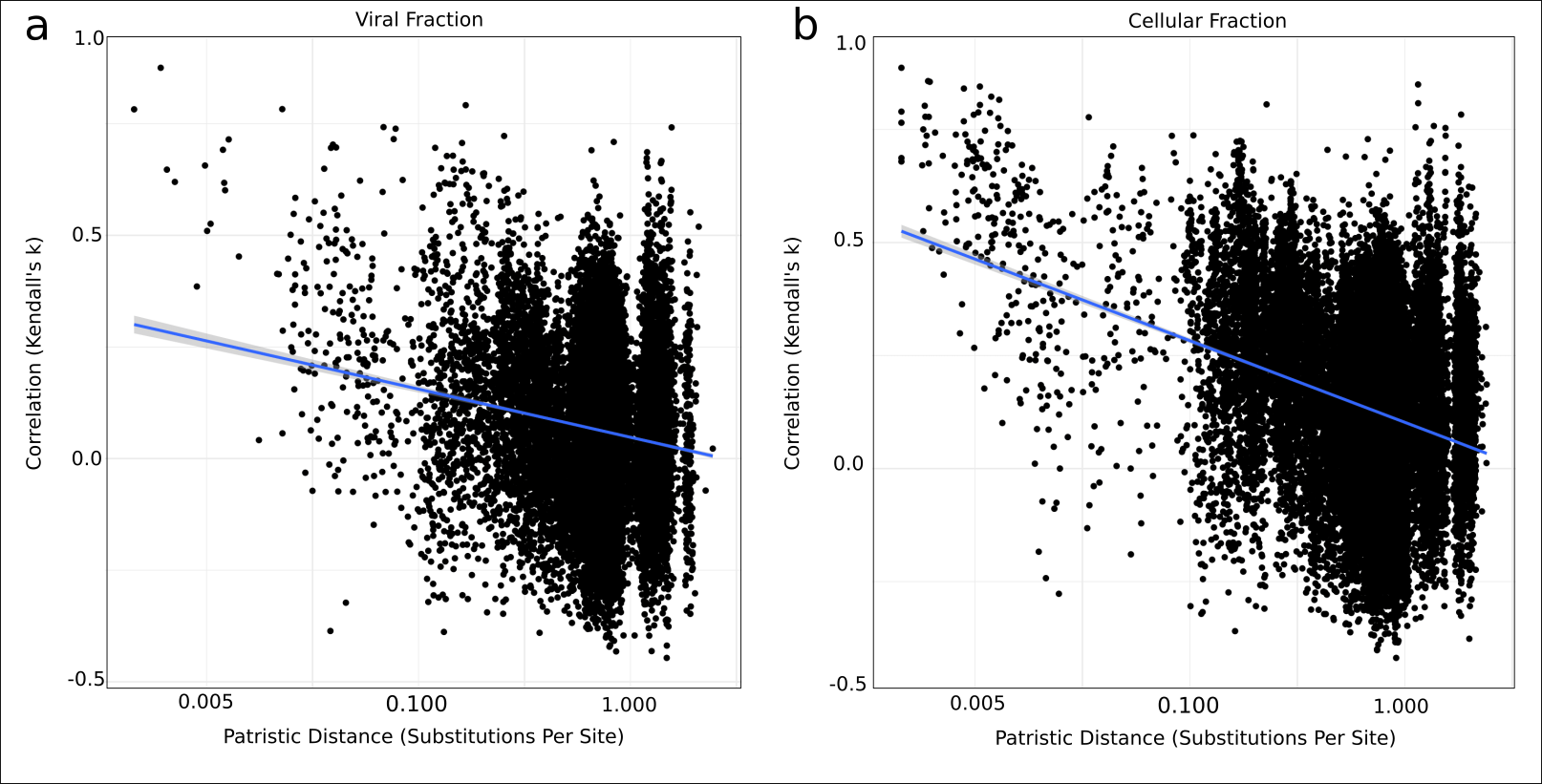


**Figure S6. Correlation and patristic distance for all viral phylotypes.** All phylotypes with abundance signal in >25% of sampled days in the (a) viral and (b) cellular fractions were considered for correlation analysis. Abundance profiles were correlated to each other using Kendall’s tau and patristic distance was calculated from phylotype trees.


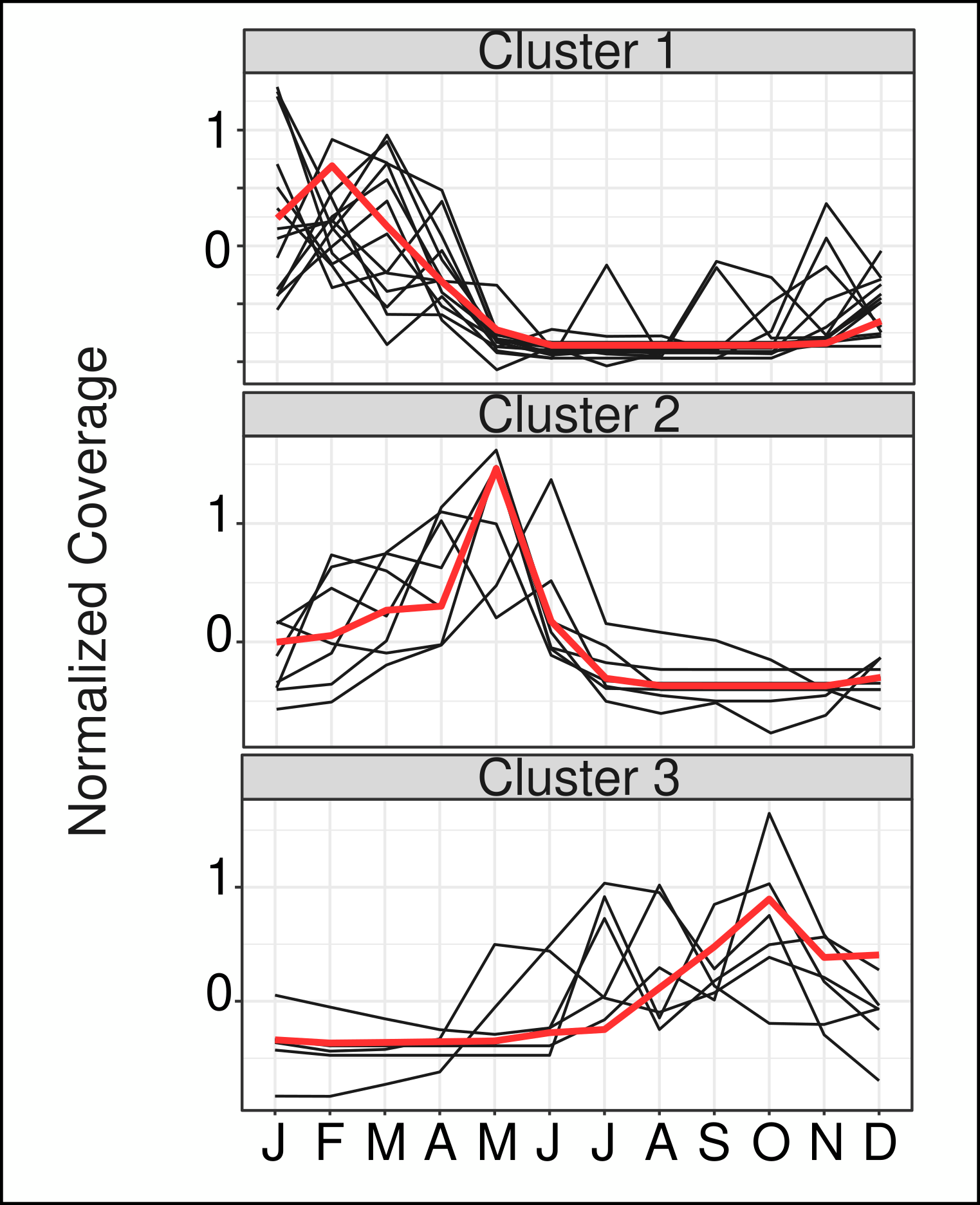


**Figure S7. 18S rRNA abundance clusters.** Clustering of 18S rRNA abundances was done using k-medoids clustering with the minimum Davies-Boudin index. Representative clusters are shown here with a red line denoting the medoid.


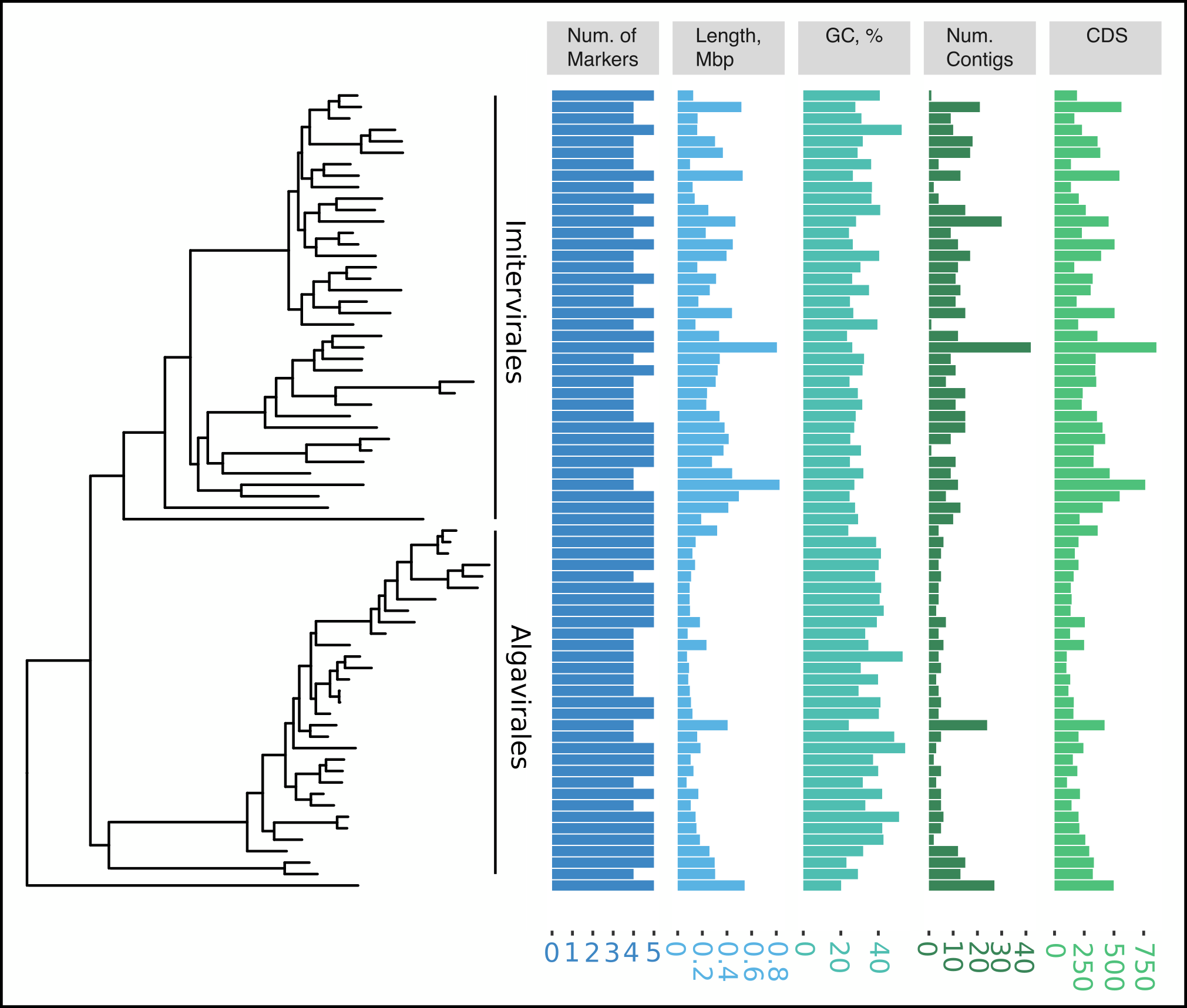


**Figure S8. Recovered NCLDV genome statistics.** The number of NCLDV marker genes, length in megabases, GC %, contig number, and coding sequences (CDS) are shown for each recovered NCLDV MAG. Phylogeny was generated as described in Figure 6.


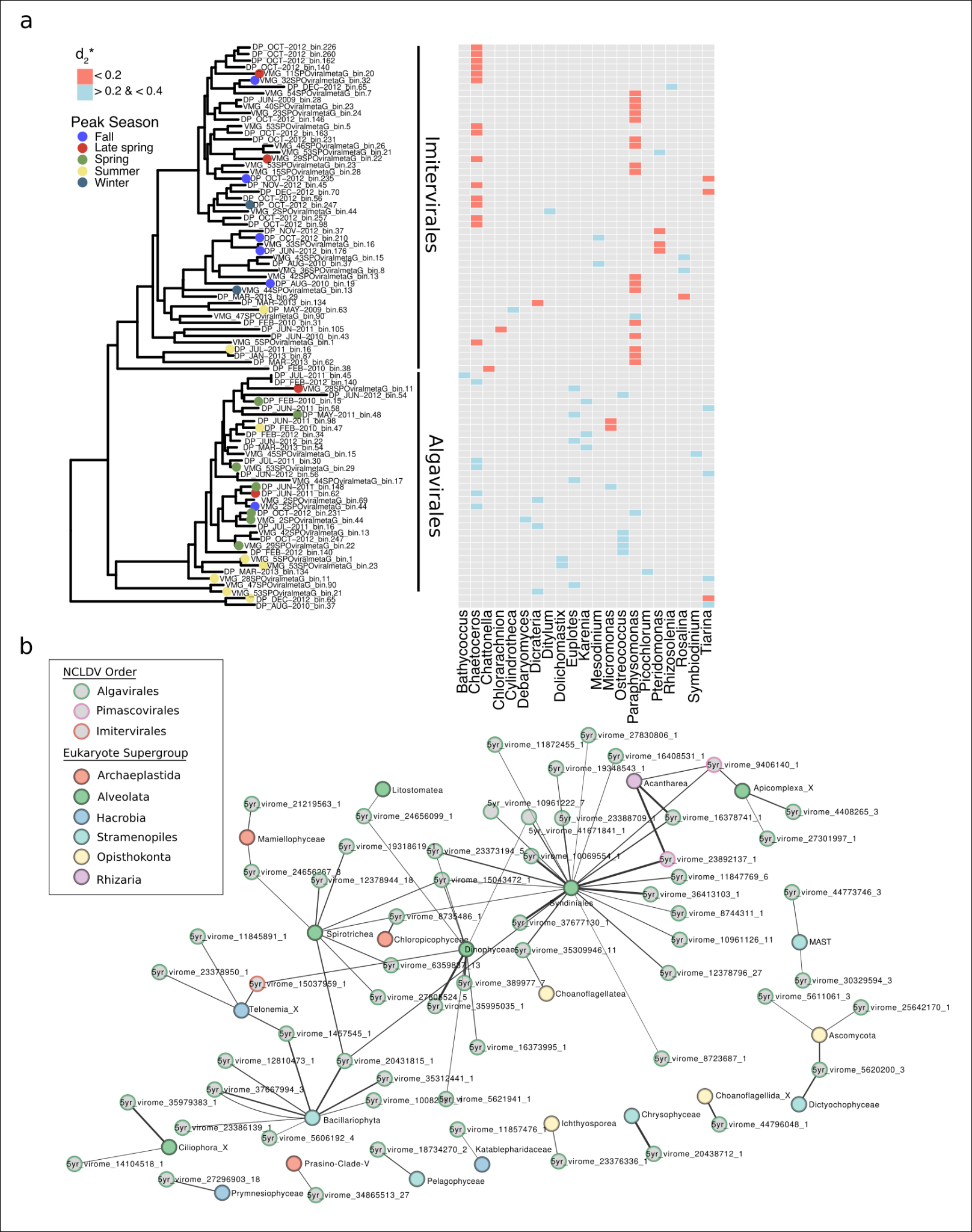


**Figure S9. Extended phylogenetic tree and network.** (a) The phylogenetic tree and host predictions from figure 7 are given leaf labels. (b) A network of host-virus interactions based on correlations between host 18S rRNA abundance and NCLDV phylotype abundance. Darker lines represent correlation strength.
